# Indicators for the evaluation of musculoskeletal trauma systems: A scoping review and Delphi study

**DOI:** 10.1371/journal.pone.0290816

**Published:** 2023-08-31

**Authors:** M. Dworkin, K. J. Agarwal-Harding, M. Joseph, G. Cahill, D. Konadu-Yeboah, E. Makasa, C. Mock, the MSK Indicator Group

**Affiliations:** 1 Department of Orthopaedic Surgery, The Warren Alpert School of Medicine at Brown University, Providence, Rhode Island, United States of America; 2 Harvard Global Orthopaedics Collaborative, Boston, Massachusetts, United States of America; 3 Department of Orthopaedic Surgery, Beth Israel Deaconess Medical Center, Harvard Medical School, Boston, Massachusetts, United States of America; 4 Global Health and Social Medicine Department, Program in Global Surgery and Social Change, Harvard Medical School, Boston, Massachusetts, United States of America; 5 Kwame Nkrumah University of Science and Technology, Kumasi, Ghana; 6 Komfo Anokye Teaching Hospital, Kumasi, Ghana; 7 Wits-SADC Regional Collaboration Centre for Surgical Healthcare, Department of Surgery, Faculty of Health Sciences, School of Clinical Medicine, University of Witwatersrand, Johannesburg, South Africa; 8 Department of Surgery, School of Medicine, University of Zambia, Lusaka, Zambia; 9 Department of Surgery, Ministry of Health, University Teaching Hospitals (UTHs), Lusaka, Republic of Zambia; 10 Department of Surgery, University of Washington, Seattle, Washington, United States of America; University of Toronto, CANADA

## Abstract

**Background:**

Trauma is a leading cause of mortality and morbidity, disproportionately affecting low- and middle-income countries. Musculoskeletal trauma results in the majority of post-traumatic morbidity and disability globally. The literature has reported many performance indicators relating to trauma care, but few specific to musculoskeletal injuries.

**Study objectives:**

The purpose of this study was to establish a practical list of performance indicators to evaluate and monitor the quality and equity of musculoskeletal trauma care delivery in health systems worldwide.

**Methods:**

A scoping review was performed that identified performance indicators related to musculoskeletal trauma care. Indicators were organized by phase of care (general, prevention, pre-hospital, hospital, post-hospital) within a modified Donabedian model (structure, process, outcome, equity). A panel of 21 experts representing 45 countries was assembled to identify priority indicators utilizing a modified Delphi approach.

**Results:**

The scoping review identified 1,206 articles and 114 underwent full text review. We included 95 articles which reported 498 unique performance indicators. Most indicators related to the hospital phase of care (n = 303, 60%) and structural characteristics (n = 221, 44%). Mortality (n = 50 articles) and presence of trauma registries (n = 16 articles) were the most frequently reported indicators. After 3 rounds of surveys our panel reached consensus on a parsimonious list of priority performance indicators. These focused on access to trauma care; processes and key resources for polytrauma triage, patient stabilization, and hemorrhage control; reduction and immobilization of fractures and dislocations; and management of compartment syndrome and open fractures.

**Conclusions:**

The literature has reported many performance indicators relating to trauma care, but few specific to musculoskeletal injuries. To create quality and equitable trauma systems, musculoskeletal care must be incorporated into development plans with continuous monitoring and improvement. The performance indicators identified by our expert panel and organized in a modified Donabedian model can serve as a method for evaluating musculoskeletal trauma care.

## Introduction

Trauma is a leading cause of mortality and morbidity worldwide causing over 5 million deaths annually and more potential years of life lost than any other illness [1, 2]. Evidence suggests that most non-fatal injuries are musculoskeletal in nature with up to 78% of trauma related to musculoskeletal injuries [3]. Most injuries occur in low- and middle-income countries (LMICs) where safe and affordable surgery as well as trauma care remain inaccessible to many [4]. Musculoskeletal injuries can have substantial economic ramifications as trauma disproportionately affects younger individuals who make up the majority of the workforce. This can push patients, families, and communities into vicious cycles of poverty [5].

Trauma-related death and disability can be averted through the implementation of comprehensive national and regional trauma systems which must include the provision of care for musculoskeletal injuries [6–10]. Poor-quality health systems are associated with over 8 million annual deaths and $6 trillion in lost economic revenue. Substandard care along with insufficient access are major barriers to reducing trauma-related mortality and morbidity [11]. It is imperative to understand quality and equity within a trauma system in order to design effective means for improvement [12]. As trauma systems worldwide develop to address the growing burden of musculoskeletal injuries, the delivery of care must be continuously monitored and improved [13].

Healthcare performance indicators (PIs) are standardized, evidence-based measures developed to compare differences between actual and ideal performance [14, 15]. PIs have been developed to assess both quality and equity of care delivery [6, 7]. Their utilization for evaluating performance and monitoring progress towards targets can improve patient outcomes as well as decrease mortality and preventable death rates [6–9]. General trauma guidelines have been developed with lists of PIs and means for their evaluation, however these general guidelines often lack specificity to comprehensively evaluate care of musculoskeletal injuries [16, 17]. The few musculoskeletal-specific indicators included in these reviews focus on use of pelvic binders for unstable pelvic ring injuries and timing of antibiotics for open fractures without examining definitive treatment or rehabilitation of these injuries. Furthermore, indicators of care equity–defined as metrics or assessments that evaluate the presence or absence of avoidable, unfair, or remediable differences in health among groups of people–are largely missing from the trauma literature [18]. Equity indicators are essential to develop comprehensive systems of care [19]. We therefore sought to establish a practical list of PIs to evaluate and monitor the quality and equity of musculoskeletal trauma care delivery in health systems worldwide.

## Methods

### Scoping review

We performed a scoping review to generate a list of PIs reported in the literature applicable to musculoskeletal trauma care. The purpose of the scoping review was to help inform a survey to be used as part of a Delphi study. A scoping review was chosen in order to identify and map the available evidence regarding the general assessment of trauma care systems as indicated by Arksey and O’Malley [20]. Musculoskeletal trauma was defined as any injury involving the extremities or spine. We sought PIs evaluating individual patient care, institutions, or health systems and encompassing all phases of care (general, prevention, pre-hospital, hospital, and post-hospital). Quality indicators were defined as metrics or assessments that evaluate the degree to which health services for individuals and populations increase the likelihood of desired health outcomes and are consistent with current professional knowledge [14, 15]. Equity indicators were metrics or assessments that evaluate the presence or absence of avoidable, unfair, or remediable differences in health among groups of people [18]. All indicators refer to the main trauma center for a given healthcare system.

**Fig 1:**
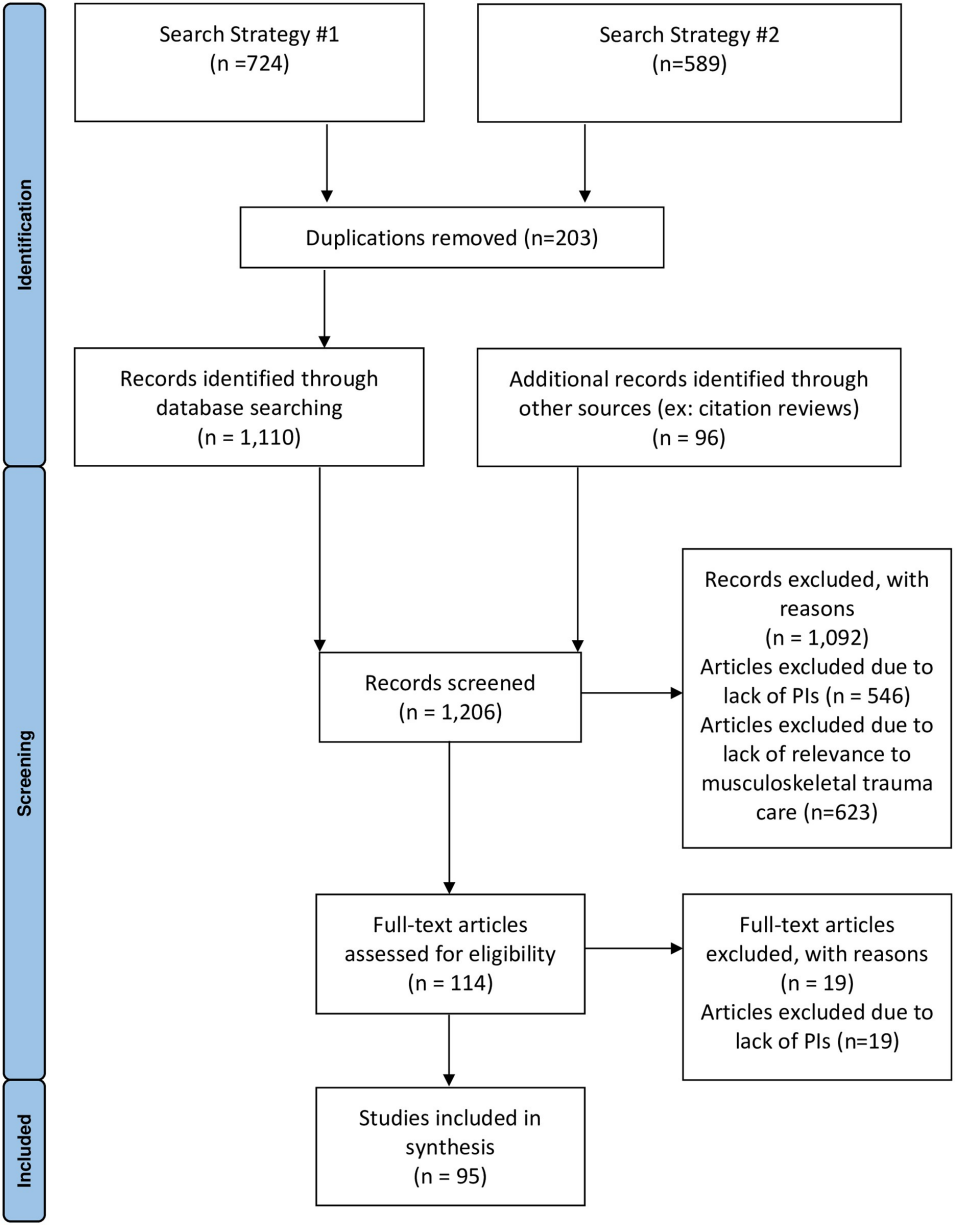
PRISMA diagram demonstrating article selection.

The methods and search strategies for this scoping review were prospectively registered with Open Science Framework [21]. A completed PRISMA-ScR can be found in S1 File. Relevant articles were identified using searches of PubMed on November 27^th^, 2019 and January 29^th^, 2020. Searches were performed with the assistance of trained librarians with no date or study design restrictions. We included articles in English, German, Spanish, and French. Our search strategies are reported in S2 File. Appropriate wildcards accounted for alternative spellings and plural words. References of included articles were also reviewed to identify additional articles for inclusion. Articles that failed to report any indicators and those that solely reported indicator not applicable to musculoskeletal care were excluded (Fig 1).

Using the web-based application Rayyan QCRI, articles underwent an initial title and abstract screening, then a full-text review by the study authors (M.D., M.J., and K.J.A.H.) [22]. Conflicts were discussed and resolved by consensus. Minimum article inclusion criteria included the identification of at least 1 PI of quality or equity, based on our previously stated definitions, that directly or indirectly related to musculoskeletal trauma care. From each paper we abstracted the country of origin, publication date, dates of data collection, study design, number of patients included, number of hospitals included, number of articles included (in the case of reviews or meta-analyses), and all PIs included.

PIs were classified according to both phase of care and the Donabedian framework as described by prior studies [16]. Phases of care included prevention, pre-hospital, hospital, and post-hospital, as well as a general category for indicators that covered multiple phases. The Donabedian framework which consists of structure, process, and outcome measures was modified to include an equity domain. After full text review of 10% of the articles, initial categories were established within the modified Donabedian model (i.e. protocols/guidelines, staff, interventions, cost/finance outcomes) to provide additional organization. These categories were reviewed and adapted iteratively as needed. Duplicates and synonymous PIs were combined with the total number of mentions recorded. All PIs and their classifications can be found in S3 File.

### Delphi study

A modified Delphi protocol and consensus survey were developed according to published guidelines for Delphi survey methodology [23]. A panel of 21 international participants with expertise in various areas of trauma care was recruited. Experts were recruited from each of the 6 World Health Organization (WHO) defined regions. Panelists included various forms of health providers such as physicians, nurses, physical and occupational therapists, and pre-hospital care providers as well as health organization leaders and policy makers. Panelists were selected through a steering committee composed of study authors (M.D., M.J., K.A.H., E.M., D.K.Y, C.M.) with additional recommendations made by panelists themselves. Requirements for participation in our panel included at least 10 years of work in a trauma related field in multiple countries and leadership in a national and/or international trauma related organization. Additional considerations were given toward academic affiliations and prior research on trauma related publications.

Panelists were provided with a series of surveys and asked to rank PIs using a 5-point Likert scale from 1 (not important) to 5 (extremely important). These scores solely represent the opinion of the experts themselves. PIs included in the survey were generated through the scoping review. Additionally, panelists were able to recommend PIs for inclusion that they felt were not identified by the scoping review. These PIs were evaluated by study authors to determine if they were significantly different than indicators previously listed. PIs within each survey were categorized by phase of care and modified Donabedian domains. We used the secure online survey tool REDCap (Research Electronic Data Capturer) [24].

In total, three Delphi rounds were completed. For the first round, panelists evaluated PIs relating to their specific field of expertise. Panelist were asked for background information and which phases of care (general, prevention, pre-hospital, hospital, and post-hospital) which they felt most comfortable performing indicator evaluation. Specific PIs for each phase of care were then assigned to individual experts. All PIs were evaluated by six panelists. Panelist reviewed indicators by assigning a Likert score to each PI with 1 being the least important and 5 being the most important. At the conclusion of the first round the scores for each PI were averaged and PIs that scored in the top tertile of scores were included in the subsequent rounds.

**Fig 2:**
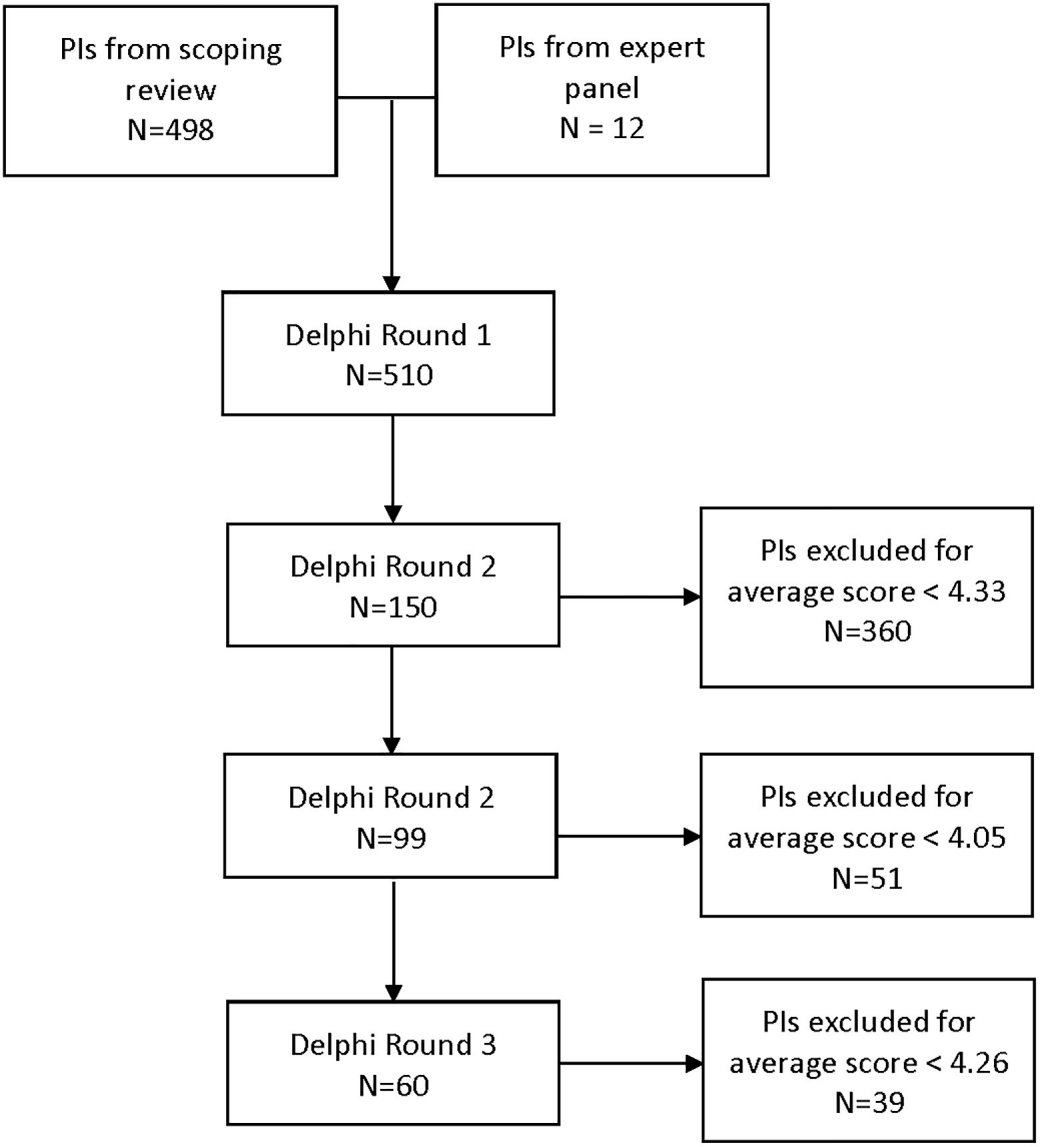
Flow diagram of the Delphi process with average score cut offs per round. For round 1, the top tertile of scores were kept. For rounds 2 and 3, the top 2 scoring tertiles of scores were kept.

Following the first round, two additional rounds were held with full review of the PIs. During these rounds each panelist evaluated all PIs that passed the previous round. We provided panelists with the mean score and standard deviation of each PI from the prior round. Panelist again assigned a Likert score to each individual PI. At the conclusion of each round the scores for an individual PI were averaged. The PIs with the lowest tertile of scores were removed (Fig 2).

Descriptive analysis was performed using SPSS version 25 (IBM Corp, Armonk, NY). The Institutional Review Board at Boston Children’s Hospital gave ethical approval for the Delphi survey methodology. No informed consent was required for this study.

## Results

### Scoping review

We identified 1,206 articles, of which 114 underwent full text review. 19 articles were excluded due to lack of PIs. A total of 95 articles, written in 4 languages (English, German, French, and Spanish), met the inclusion criteria (Fig 1). The most common study design was a cohort study (n = 28, 29%) followed by narrative reviews and systematic reviews (n = 16, 17%). Articles were from 29 countries with 18 (16%) studies originating from LMICs. No articles provided PIs exclusively pertaining to musculoskeletal injuries, but rather all articles examined trauma systems or health systems more generally.

From the 95 included articles, we identified 1,231 PIs related to quality or equity of musculoskeletal trauma care delivery. Duplicate and synonymous indicators were combined yielding 498 unique PIs (S3 and S4 Files).

The most mentioned PIs were general trauma system-wide mortality (n = 35 articles, 37%), presence of a trauma registry (n = 16, 17%), in-hospital mortality/survival (n = 15, 16%), presence of a lead agency in charge of the trauma system (n = 14%), and implementation of a formalized trauma system that provides a continuum of services (n = 14%) (Table 1). There were 67 (12.5%) PIs that were musculoskeletal-specific including metrics such as the number of orthopedic surgeons and non-physician providers working in a country and the availability of orthopedic trauma fellowship training (Table 1).

**Table 1:** Ten most frequently mentioned performance indicators and musculoskeletal-specific indicators.

Indicator	Donabedian Model	Phase of Care	Number of Mentions
All Indicators			
Trauma System-Wide Mortality	General	Outcome	35
Trauma Registry	General	Structure	16
In-Hospital Mortality/Survival	Hospital	Outcome	15
Lead Agency in Charge of Trauma System	General	Structure	13
Organized/Formalized Trauma System	General	Structure	13
Adverse Events/Complications	Hospital	Outcome	12
Formal Accreditation of Trauma Facilities	Hospital	Structure	12
Prehospital Time (Total)	Prehospital	Process	12
Access to Rehabilitation	Posthospital	Structure	11
Trauma trained prehospital staff	Prehospital	Structure	11
Musculoskeletal-Specific Indicators			
Quantity of Orthopaedic Surgeons	Hospital	Structure	5
Quantity of Non-Physician Orthopaedic Clinical Officers	Hospital	Structure	4
Trauma Fellowship Training	Hospital	Structure	4
Immobilization of Suspected Spine Injuries	Hospital	Process	4
Time to Surgery of Open Fractures	Hospital	Process	4
Ability to Management and Care for Amputations	Hospital	Process	4
Management of Closed Fractures	Hospital	Process	4

According to our modified Donabedian framework, most indicators pertained to *Structure* (n = 221, 44%) followed by *Process* (n = 128, 23.5%), *Equity* (n = 99, 18.0%), then *Outcomes* (n = 84, 16.0%). The majority of indicators referred to the hospital phase of care (n = 303, 60%), followed by general system-wide trauma care (n = 109, 20%) (Table 2). An expanded classification of indicators can be seen in S5 File.

**Table 2:** Classification of unique performance indicators identified in study.

	General	Prevention	Pre-Hospital	Hospital	Post-Hospital	Total
Structure	37	8	22	152	12	221
Process	9	3	23	91	2	128
Outcome	13	1	2	42	26	84
Equity	50	13	5	28	3	99
Total	109	25	52	303	43	498

### Delphi study

We contacted 24 potential panelists for participation in the Delphi study of which 21 (87.5%) agreed to inclusion. The 21 expert panelists had professional backgrounds in orthopedic surgery (n = 12), general trauma surgery (n = 4), emergency medicine (n = 2), nursing (n = 2), and physical therapy (n = 1). All World Health Organization regions were represented by panelists: the Americas (n = 10, 48%), Africa (n = 5, 24%), South-East Asia (n = 2, 9.5%), Europe (n = 2, 9.5%), Eastern Mediterranean (n = 1, 5%), and Western Pacific (n = 1, 5%). Furthermore, panelists reported professional experience in 46 countries. Surveys 1 and 2 were completed by all panelists, survey 3 by 19 (90.5%) panelists.

The expert panel identified a list of high priority indicators. Most PIs referred to the hospital phase of care (n = 45, 75%) followed by prehospital (n = 9, 15%) and general trauma system wide indicators. (n = 6, 10%). The most common Donabedian category was structural (n = 32, 53%) followed by process indicators (n = 21, 35%).

The list of PIs focused on access to trauma care (n = 4, 7%); processes and key resources for polytrauma triage (n = 28, 46%), patient stabilization (n = 5, 8%), and hemorrhage control (n = 5, 8%); reduction and immobilization of fractures and dislocations (n = 6, 10%); and management of compartment syndrome (n = 1, 2%) and open fractures (n = 5, 8%). Examples of access metrics included percentage of the population with access to prehospital care and orthopedic trauma surgeons. Critical resources included basic utilities such as electricity, oxygen, and fluids for resuscitation, as well as specific supplies like skeletal traction and external fixation equipment. Panelists additionally emphasized several process indicators such as the timely ability to manage common musculoskeletal injuries and emergencies. Examples included time from identification of compartment syndrome to fasciotomy and the reduction of dislocations within 1 to 4 hours. A summary of indicators recommended by our expert panel can be seen in Table 3. A complete list of indicators with scores can be seen in S6 File.

**Table 3:** Final list of high priority indicators identified by our expert panel.

	General Trauma System Performance
Structure	Is Trauma included in the national health plan?
Process	Does regular or continuous monitoring and evaluation of trauma system performance occur? What is the average time from injury to surgery for musculoskeletal trauma patients?
Outcome	What are the total number of preventable trauma-related deaths?
Equity	What percentage of the population has access to formal trauma care regardless of ability to pay? Rates of catastrophic or impoverishing expenditures for trauma care. What percentage of the population has access to essential medications*? What percentage of the population has access to essential resources*?
	Pre-Hospital Trauma System Performance
Structure	For the following essential components of care: Basic Life Support Airway management Hemorrhage control Immobilization Are protocols in place for management in the pre-hospital setting? Are essential resources available (splints/slings, cervical collars, back boards, tourniquets, airway management supplies)?
Process	For the following essential components of care: Basic Life Support Airway management Hemorrhage control Immobilization Are pre-hospital staff well-trained and capable?
Outcome	Pre-hospital mortality rate
Equity	What percentage of the population has timely access to formal pre-hospital care*?
	Hospital
Structure	What percentage of hospitals have basic infrastructure (stable electricity with back-up generator, running water, oxygen)? What percentage of hospitals have availability of safe anesthesia? General anesthesia essential equipment and medications (incl. oxygen, ventilators, inhalational anesthesia) Regional anesthesia essential equipment and medications Anesthesia and pain management guidelines (incl. for sedation and preoperative risk assessment) What number/percentage of hospitals have availability of the following diagnostics: Formal x-ray Intra-operative fluoroscopy What percentage of hospitals have the following essential resources available: Examination gloves Tetanus vaccination Pain medication Antibiotics Means for sterilization of equipment What percentage of hospitals have the following essential procedures available (including resources, skilled staff, infrastructure, and protocols): Stabilization and monitoring of trauma patient Circulation and shock management (hemorrhage identification and control, chest tubes, IV fluids, infusion sets, blood products, pressors) Irrigation and debridement of wounds and burns Immobilization equipment (splints, slings, pelvic binders, skeletal traction, cervical collars) Rehabilitation equipment (crutches, walkers) What percentage of hospitals have the following components of open fracture management available (including resources, skilled staff, infrastructure, and protocols): Wound management Equipment sterilization Fracture external fixation Fracture internal fixation Flap coverage
Process	Average time from identification of compartment syndrome to fasciotomy Average time from arrival to antibiotics (within 1hr) / surgery for open fractures Average time from arrival to reduction of major joint dislocations Time to hemorrhage control Time from arrival to securing an airway Time from arrival to vital signs measurement/monitoring (within 15 mins). Time from arrival to pelvic binder placement for suspected unstable pelvic injuries. Time from arrival to operating room for patient with life threatening injury requiring surgery
Outcome	Mortality due to hemorrhagic shock Mortality/survival rate in pts with serious injury (ISS > 15)
Equity	What percentage of the population has timely access to orthopaedic trauma care?

*Please see pre-hospital and hospital tables for specifics medications and resources.

## Discussion

Musculoskeletal trauma is responsible for the majority of global disability and non-fatal injuries [6]. Unfortunately, trauma systems in many countries remain unable to provide high quality equitable care for these injuries [25]. We sought to identify key components of musculoskeletal trauma care and propose a means for their evaluation. Our scoping review of the literature identified 498 unique PIs relevant to the evaluation of musculoskeletal trauma care delivery. Through a Delphi survey, our expert panelists established a list of high priority PIs. These indicators provide a practical guide to evaluating and monitoring the quality and equity of musculoskeletal trauma care delivery in health systems around the world. Essential aspects included timely access to well-equipped trauma systems, initial evaluation and resuscitation of trauma patients, and appropriate care of musculoskeletal emergencies.

Key PIs related to access included percentage of the population with availability of pre-hospital, emergency, and orthopaedic care. There are many examples of how increasing access to trauma care improves patient outcomes. In Monterey, Mexico, strategically distributing ambulance dispatch stations throughout the city increased the percentage of the population with access to pre-hospital care and reduced mortality from 8.2% to 4.7% [26]. At the main trauma hospital in Khon Kaen, Thailand, initiatives that increased staffing within their emergency department improved timely access to providers and helped reduce trauma mortality from 6.1% to 4.4% [27]. In the United States, the implementation of the Affordable Care Act reduced the number of uninsured Americans which has been found to be associated with greater access to trauma services and reduced financial risk [28]. In addition to accessing trauma centers, our panelists identified key resources, the presence of which signals an ability to provide adequate musculoskeletal trauma care. This ranged from basics such as oxygen, running water, and electricity to more specific supplies including external fixation, skeletal traction, and diagnostic x-ray. These findings are consistent with the resources described by the WHO’s Guidelines for Essential Trauma Care as well as the AO Aliance [29, 30]. Management of these resources is also essential as breakdown necessitating repair is common [31]. Medical equipment donations to LMICs are common but require equitable partnerships and careful planning to increase usability and lifespan. Appropriate procurement, allocation, and replenishment of essential resources improves access and reduces complications.

The recommendations of our expert panel brought attention to the critical role of the initial evaluation, triage, and resuscitation of all trauma patients. Specific PIs related to initial management included time to hemorrhage control, application of pelvic binders for patients with suspected unstable pelvic fractures, immobilization of suspected spinal injuries and presence of standard life support protocols such as the Advanced Trauma Life Support training program. All of these are proven methods for reducing mortality [32, 33]. For example, hemorrhage remains responsible for 30–40% of trauma-related deaths with many occurring in the prehospital setting. The implementation of bystander interventions aimed at reducing time to hemorrhage control have been effective in a number of HIC and LMIC settings [34–36]. Inclusion of PIs covering initial trauma management highlights the need for integrating musculoskeletal trauma care into comprehensive trauma systems as well as the incorporation of general trauma principles in orthopaedic training.

PIs specifically related to musculoskeletal trauma focused on common injuries (ex. the number or percentage of hospitals in country able to perform fracture immobilization; time from hospital arrival to joint reduction) as well as emergencies (ex. the availability of antibiotics for open fractures; time from the diagnosis of compartment syndrome to fasciotomy). These indicators have proven to be useful in the literature both in terms of identifying problems and designing targeted interventions. For example, a hospital in Malawi reported unacceptably high rates of infections following open tibial shaft fractures. This was related to challenges with the provision of antibiotics. In response, a standardized protocol was developed emphasizing early antibiotic administration and sequential debridement. Study outcomes revealed no long-term infections and excellent functional outcomes in 80% of patients [37]. Another study surveyed 883 hospitals in 24 LMICs and found that only about 30% of facilities were able to perform closed management of fractures while 43% were capable of reducing major joint dislocations [38]. This led to calls for reallocation of resources. Systematic implementation of these PIs into regular monitoring and evaluation of trauma systems could help identify deficiencies and plan interventions to address them.

The PIs identified in our study were organized using a modified Donabedian model. The classic Donabedian framework suggests that health care quality can be evaluated in terms of structure, process, and outcome metrics and has been validated within general trauma systems [39]. We proposed the additional inclusion of equity into this model to gain a more comprehensive understanding of a system’s performance. It is well-established that factors such as education, income level, and geographical availability of care can impact health outcomes as can other social determinants of health [40]. Moreover, disparities in disease burden, care access, and outcomes have been demonstrated in low-, middle-, and high-income countries [41]. Our literature review found few studies which included equity indicators when evaluating trauma care. Trauma-related equity indicators made up 16% of all PIs in the literature and 10% of PIs identified by our expert panel. We believe that equity should be considered alongside quality when assessing the performance of trauma systems and further development and validation of equity indicators is needed.

This study proposes a list of indicators as a practical guide to evaluate and monitor musculoskeletal trauma care delivery as a part of effective trauma systems in low-, middle-, and high-income countries. This study had a number of limitations. We performed a scoping review to identify PIs from the literature. As such, risk of bias of the evidence was not performed. Furthermore, only two search strategies of a single database were utilized. This was done to facilitate a more thorough citation review which we felt would be more beneficial for identifying relevant articles. We additionally encouraged panelist from our Delphi study to propose indicators that they felt were missing. These indicators were then used to populate the Delphi surveys. Although the number of included indicators is similar to previous work evaluating general trauma systems, it is likely that our lists are not entirely comprehensive [12]. For example, the final list of PIs created by our panel did not include indicators regarding injury prevention or post-hospital rehabilitation despite several reported in the literature. Similarly, specific PIs related to geriatric trauma were not included. Furthermore, aside from the inclusion of spinal immobilization, there were no indicators regarding spinal trauma. Despite these exclusions, we feel the proposed list represents a series of bellwether indicators that may provide a review of the overall function of a musculoskeletal trauma system. Additionally, our panel was composed of providers with expertise in different areas of trauma care and professional experience in diverse settings around the world. However, our results were subject to their discretion and did not specifically take into consideration evidence from the literature. As such the results may not be generalizable to all settings. The final list of PIs solely represents the opinions of our expert panel and does not include rationale for why certain indicators were ranked higher than others. The final prioritized list included the highest scoring PIs from several rounds of surveys, but many of the PIs eliminated may merit further consideration. It should also be noted that while it is useful to organize PIs within our proposed categories, most PIs are overlapping and involve interactions throughout the spectrum of care. We have not yet tested these indicators to determine feasibility of data collection or clinical relevance, however, future work is planned to validate these measures.

Similar list including the American College of Surgeon’s Regional Trauma Systems Report and the WHO’s Guidelines for Essential Trauma Care are also available. These prior publications contain many trauma system characteristics included in our final list such as the need for national planning, the importance of continuous evaluation for quality improvement, and the requirement of key resources. These reports improve trauma care understanding and provide valuable resources for systems development. Our list adds to this conversation and provides important insight into means for evaluation. Our list presents actionable indicators related to various aspect of musculoskeletal care within a straightforward framework that is beneficial for monitoring performance.

In conclusion, we propose a list of high priority PIs related to musculoskeletal trauma care delivery with key indicators related to timely access to well-equipped trauma systems, initial evaluation and resuscitation of trauma patients, and appropriate care of musculoskeletal emergencies. These PIs organized within a modified Donabedian model can be used to optimize resource and service allocation, perform national audits to track system progress, and provide international standards for evaluation. Musculoskeletal injuries must be considered in the development of trauma systems to effectively prevent unnecessary death, disability, and destitution.

## Supporting information

S1 File Preferred Reporting Items for Systematic reviews and Meta-Analyses extension for Scoping Reviews (PRISMA-ScR) checklist. (DOCX)

S2 File Database search strategies. (DOCX)

S3 File All indicators organized by phase of care and Donabedian category. (DOCX)

S4 File List of articles included in scoping review as well as corresponding indicators obtained from each article. (XLSX)

S5 File Expanded classification of unique performance indicators identified in study. (DOCX)

S6 File Results of each round of the Delphi survey with scores for each included indicator. (XLSX)
