# Supplementary material for: Indicators for the evaluation of musculoskeletal trauma systems: A scoping review and Delphi study
Source: PLoS One. 2023 Aug 31;18(8):e0290816. doi: 10.1371/journal.pone.0290816 (PMC10470913; doi:10.1371/journal.pone.0290816)
Supplement: S2 File — (DOCX) [file pone.0290816.s002.docx]

Supporting Information 2: Database search strategies.

|  |  |  |
| --- | --- | --- |
| Search #1 Terms | Date Search Performed | Results |
| (((Systems[Title]) OR Indicators[Title] OR Musculoskeletal Injuries[Title] OR Orthopaedic Trauma[Title])) AND ((((((((((("Quality Indicators, Health Care"[Mesh]) OR ("Health Care Quality, Access, and Evaluation"[Mesh])) OR "Quality of Health Care"[Mesh]) OR "Health Care Evaluation Mechanisms"[Mesh]) OR "Program Evaluation"[Mesh]) OR "Quality Improvement"[Mesh])) AND (((("Efficiency, Organizational"[Mesh]) OR "Health Services Research"[Mesh]) OR "Population Health Management"[Mesh]) OR "Delivery of Health Care"[Mesh])) AND ((((("Trauma Centers"[Mesh]) OR ("Wounds and Injuries"[Mesh])) OR "Traumatology"[Mesh]) OR "Emergency Medical Services"[Mesh]) OR Surgical Traumatology)))) | 11/27/2019 | 724 |
|  | | |

| Search #2 Terms | Date Search Performed | Results |
| --- | --- | --- |
| ("Fractures, Bone"[Mesh] OR "Joint Dislocations"[Mesh] OR dislocation*[tiab] OR fracture[tiab] OR fractures[tiab] OR (musculoskeletal[tiab] OR skeletal[tiab] orthopaedic[tiab] OR orthopedic) AND (trauma[tiab] OR traumatic[tiab] OR injur*)) AND (affordable[tiab] OR affordability[tiab] OR ((access[tiab] OR accessible[tiab] OR accessibility[tiab] OR availability[tiab] OR disparity[tiab] OR disparities[tiab]) AND (emergency care[tiab] OR health care[tiab] OR healthcare[tiab] OR trauma care[tiab])) OR catastrophic expenditure*[tiab] OR equality[tiab] OR equity[tiab] OR financial risk protection[tiab] OR health system*[tiab] OR impoverishing expenditure*[tiab] OR inequity[tiab] OR out of pocket [tiab] OR unaffordable[tiab] OR universal health care[tiab]) | 1/29/2020 | 589 |
