# Supplementary material for: Indicators for the evaluation of musculoskeletal trauma systems: A scoping review and Delphi study
Source: PLoS One. 2023 Aug 31;18(8):e0290816. doi: 10.1371/journal.pone.0290816 (PMC10470913; doi:10.1371/journal.pone.0290816)
Supplement: S3 File — (DOCX) [file pone.0290816.s003.docx]

Supporting Information 3: All indicators organized by phase of care and Donabedian category.

| **Indicator** | **Phases of care** | **Donabedian** | **Category** | **Mentions** |
| --- | --- | --- | --- | --- |
| Density/distribution of trauma center and acute care facilities (esp. rural) | General | Equity | Access | 5 |
| Health insurance coverage | General | Equity | Access | 5 |
| Healthcare utilization | General | Equity | Access | 4 |
| Access to emergency care and resources regardless of ability to pay | General | Equity | Access | 2 |
| Access to a core set of relevant essential medicines | General | Equity | Access | 1 |
| Availability of universal healthcare | General | Equity | Access | 1 |
| Community understanding or knowledge of services | General | Equity | Access | 1 |
| Health Utilities Index | General | Equity | Access | 1 |
| Number of health facilities offering specific services per 10000 population (basic surgery, pediatric care, blood transfusions, laboratory capacity) | General | Equity | Access | 1 |
| Incidence of major trauma (ISS > 15) | General | Equity | Burden | 2 |
| Incidence/No. of injuries (national and regional) | General | Equity | Burden | 2 |
| % of injuries due to RTI | General | Equity | Burden | 1 |
| % of injuries resulting in extermity trauma | General | Equity | Burden | 1 |
| Frequency rates of occupational injuries | General | Equity | Burden | 1 |
| Incidence of geriatric trauma | General | Equity | Burden | 1 |
| Incidence of treated and untreated fractures/100,000 | General | Equity | Burden | 1 |
| Intimate partner violence prevalence | General | Equity | Burden | 1 |
| Mortality rate due to homicide | General | Equity | Burden | 1 |
| Non-partner sexual violence prevalence | General | Equity | Burden | 1 |
| Number of deaths, missing persons and persons affect by diaster per 100,000 people | General | Equity | Burden | 1 |
| Age | General | Equity | Demographics | 9 |
| Race/ethnicity (ethnic minorities) | General | Equity | Demographics | 7 |
| Population (total, density, % urban/rural) | General | Equity | Demographics | 6 |
| Gender | General | Equity | Demographics | 5 |
| Education level | General | Equity | Demographics | 2 |
| Citizenship | General | Equity | Demographics | 1 |
| Occupation | General | Equity | Demographics | 1 |
| Workforce per 100,000 population | General | Equity | Demographics | 1 |
| Financial risk (incl. rates of and protection against catastrophic and impoverishing expenditures, esp for surgical and anesthesia care) | General | Equity | Economics | 10 |
| Socioeconomic status (measured as sponsor rank) | General | Equity | Economics | 7 |
| GDP (total, per capita) | General | Equity | Economics | 3 |
| Government vs private expenditure on health care as % of total spending | General | Equity | Economics | 2 |
| Public domestic vs external sources of current spending on health as % of current health expenditure | General | Equity | Economics | 2 |
| Total annual health expenditure as % of GDP | General | Equity | Economics | 2 |
| % of population below international poverty line | General | Equity | Economics | 1 |
| Copays, out-of-pocket expenditures on health | General | Equity | Economics | 1 |
| Difference between riches and poorest | General | Equity | Economics | 1 |
| Human Development Index Rank | General | Equity | Economics | 1 |
| Total net official development assistance to medical research and basic health sectors | General | Equity | Economics | 1 |
| United Nations Development Index | General | Equity | Economics | 1 |
| General population mortality (by age groups) | General | Equity | Non-trauma medical | 9 |
| Comorbidities (hypertension, diabetes, obesity, mental health) | General | Equity | Non-trauma medical | 8 |
| Nutrition (Children under 5 who are stunted, wasted, overweight; Incidence of low birth weight among newborns) | General | Equity | Non-trauma medical | 7 |
| Non-trauma mortality (Premature noncommunicable, Maternal) | General | Equity | Non-trauma medical | 4 |
| Risk factors (Alcohol use per capita (15+), tobacco use per capita (15+), BMI) | General | Equity | Non-trauma medical | 4 |
| Anaemia prevalence in children, women of reproductive age | General | Equity | Non-trauma medical | 2 |
| C-section rate | General | Equity | Non-trauma medical | 2 |
| Life expectancy at birth | General | Equity | Non-trauma medical | 2 |
| Tuberculosis incidence and prevalence per 100 000 inhabitants | General | Equity | Non-trauma medical | 2 |
| HIV prevalence among adults per 100 000 inhabitants | General | Equity | Non-trauma medical | 1 |
| Mortality (trauma-related, #/%) | General | Outcome | Clinical Outcome | 35 |
| DALYs (YLLs, YLDs) (total, per 100,000 population) | General | Outcome | Clinical Outcome | 4 |
| Morbidity (trauma-related) (#,%) | General | Outcome | Clinical Outcome | 2 |
| Mortality (road traffic, #/%) | General | Outcome | Clinical Outcome | 2 |
| Death Incidence rates | General | Outcome | Clinical Outcome | 1 |
| Morbidity by mechanism (road, industry, farming, sport accidents) (#,%) | General | Outcome | Clinical Outcome | 1 |
| Mortality by mechanisms (#,%) | General | Outcome | Clinical Outcome | 1 |
| Total Cost | General | Outcome | Cost/Finance | 2 |
| Cost-effectiveness | General | Outcome | Cost/Finance | 2 |
| Cost needed to eliminate burden of averatble incidents DALYs | General | Outcome | Cost/Finance | 1 |
| Economic impact of injury | General | Outcome | Cost/Finance | 1 |
| Presence of user feeds | General | Outcome | Cost/Finance | 1 |
| Private vs. public care | General | Outcome | Cost/Finance | 1 |
| The trauma system engages in regular/continuous monitoring and evaluation of trauma system performance | General | Process | Quality Improvement | 5 |
| Laws, rules, and regulations are routinely reviewed and revised | General | Process | Quality Improvement | 1 |
| Presence of performance improvement programs | General | Process | Quality Improvement | 1 |
| The trauma system evaluation involves independent external reviews | General | Process | Quality Improvement | 1 |
| Femur fracture treated within 48 hrs | General | Process | Quality of Care | 1 |
| National whole blood donation rate | General | Process | Quality of Care | 1 |
| Time from injury to being seen by orthopaedic specialist | General | Process | Quality of Care | 1 |
| Time from injury to surgery | General | Process | Quality of Care | 1 |
| Use of ACS standards for trauma centers | General | Process | Quality of Care | 1 |
| Provider education | General | Structure | Education | 2 |
| Leadership committee for education | General | Structure | Education | 1 |
| Output of training institutions | General | Structure | Education | 1 |
| Population education and outreach (prevention, health literacy) | General | Structure | Education | 1 |
| Presence of ATLS program | General | Structure | Education | 1 |
| Trauma registry | General | Structure | Organization/Component | 16 |
| Lead agency in charge of trauma system (trauma registry, supply chain management, monitoring/evaluation of performance and costs) | General | Structure | Organization/Component | 13 |
| Organized/formalized trauma system (provides a continuum of services including prevention programs, prehospital care, acute care, and rehabilitation) | General | Structure | Organization/Component | 13 |
| Funding of trauma system (planning, implementation, and ongoing management of administrative and clinical care components) | General | Structure | Organization/Component | 4 |
| Organized/formalized network of trauma centers | General | Structure | Organization/Component | 4 |
| Diaster planning and management | General | Structure | Organization/Component | 3 |
| Government agency involved in trauma system | General | Structure | Organization/Component | 3 |
| Age/maturity of trauma system | General | Structure | Organization/Component | 2 |
| Evidence of regionalised trauma care (not necessarily national) | General | Structure | Organization/Component | 2 |
| Government funds trauma system | General | Structure | Organization/Component | 2 |
| Lead agency has sufficient legal authority to ensure and enforce compliance (accredidation) | General | Structure | Organization/Component | 2 |
| Lead agency works in concert with a trauma-specific multidisciplinary, multiagency advisory committee | General | Structure | Organization/Component | 2 |
| Birth registration | General | Structure | Organization/Component | 1 |
| Completeness of registry reporting by facilities | General | Structure | Organization/Component | 1 |
| Corruption | General | Structure | Organization/Component | 1 |
| Death registration | General | Structure | Organization/Component | 1 |
| Existence of linked registries (crash data, ambulance service, data, administrative and discharge data, rehab, coroner findings, insurance data, long-term disability data) | General | Structure | Organization/Component | 1 |
| Existence of registries (crash data, ambulance service, data, administrative and discharge data, rehab, coroner findings, insurance data, long-term disability data) | General | Structure | Organization/Component | 1 |
| Health provider associations or professional groups focused on trauma | General | Structure | Organization/Component | 1 |
| Trauma included in national health plan | General | Structure | Organization/Component | 1 |
| Trauma systems are to be exclusive, treating seriously injured patients in a restricted number of designated trauma centers | General | Structure | Organization/Component | 1 |
| Type of system (public, private, mixed) | General | Structure | Organization/Component | 1 |
| Standardized national guidelines and protocols for trauma care systems | General | Structure | Protocols/Guidelines | 4 |
| Distaster protocols and surge capacity | General | Structure | Protocols/Guidelines | 1 |
| Legislation to improve access and standardization | General | Structure | Protocols/Guidelines | 1 |
| Trauma research and scholarship | General | Structure | Research | 5 |
| National trauma research action plan and funding | General | Structure | Research | 1 |
| Essential resources | General | Structure | Resources | 1 |
| Resources for trauma team and trauma service organization | General | Structure | Resources | 1 |
| Health worker density and distribution | General | Structure | Staff | 1 |
| Human resources | General | Structure | Staff | 1 |
| Presence of task sharing | General | Structure | Staff | 1 |
| Geographic and climate factors to accessibilty of surgical facilities (living distance from trauma center) | Hospital | Equity | Access | 5 |
| Outpatient healthcare utilization at 30/90 days | Hospital | Equity | Access | 4 |
| Coverage area of trauma centers | Hospital | Equity | Access | 3 |
| Number of people served by trauma center (recommended 1 million per level 1 center) | Hospital | Equity | Access | 3 |
| Access to the trauma center within 1, 2,and 4 hrs/85 minutes of injury | Hospital | Equity | Access | 2 |
| Population served by health facility | Hospital | Equity | Access | 2 |
| % of patients transfered to trauma center vs. direct admits | Hospital | Equity | Access | 1 |
| % of population with access to safe and affordable surgical care | Hospital | Equity | Access | 1 |
| Access of the seriously injured to designated trauma centers | Hospital | Equity | Access | 1 |
| Access to orthopaedic trauma care, <1h, <2h | Hospital | Equity | Access | 1 |
| Adequate coverage of trauma centers | Hospital | Equity | Access | 1 |
| Adequate orthopaedic trauma access | Hospital | Equity | Access | 1 |
| Hospital location (urban vs. rural) | Hospital | Equity | Access | 1 |
| Proportion of the population that can access, within 2 hours, a facility that can do cesarean delivery, laparotomy, and treatment of open fracture (the bellwether procedures) | Hospital | Equity | Access | 1 |
| Surgical systems: Access to timely essential surgery | Hospital | Equity | Access | 1 |
| Trauma centers in remote areas | Hospital | Equity | Access | 1 |
| # of trauma surgeries performed annually (per 100,000 population, volume) | Hospital | Equity | Burden | 5 |
| Hospital admissions due to truama annually | Hospital | Equity | Burden | 4 |
| Injury severity classification (ISS, TRISS, RTS) | Hospital | Equity | Burden | 3 |
| Annual cases of major trauma (ISS > 15) | Hospital | Equity | Burden | 2 |
| Injury type and number of cases | Hospital | Equity | Burden | 2 |
| % of severely injured pts (ISS > 15) that underwent surgery during hospital stay | Hospital | Equity | Burden | 1 |
| % of severely injured pts admitted to ICU (ISS > 15) | Hospital | Equity | Burden | 1 |
| % of emergency department visits due to injury | Hospital | Equity | Burden | 1 |
| Hospital population coverage | Hospital | Equity | Burden | 1 |
| Number of orthopaedic surgeries per year | Hospital | Equity | Burden | 1 |
| Orthopaedic trauma patients per year | Hospital | Equity | Burden | 1 |
| Referral population area | Hospital | Equity | Burden | 1 |
| Mortality/Survival (in hospital #/rate, expected/observed, risk-adjusted, standardized ratio) | Hospital | Outcome | Clinical Outcome | 15 |
| Adverse events/Complications (DVT, pulmonary embolism, shock, hemorrhage, myocardial infarction, renal failure, respiratory failure, neurologic complication, sepsis, decubitus ulcer | Hospital | Outcome | Clinical Outcome | 12 |
| Readmission rates (30 day) | Hospital | Outcome | Clinical Outcome | 6 |
| Reintubation (within < 12/48 hrs, ACCIDENTAL!) | Hospital | Outcome | Clinical Outcome | 5 |
| Unplanned return to OR (within 24-48hrs, 30 days) | Hospital | Outcome | Clinical Outcome | 4 |
| Morality Perioperative rate (#, 30-day perioperative) | Hospital | Outcome | Clinical Outcome | 4 |
| Fractures reduction success (no readmissions or future operative fixation required, error in technique, un-planned reoperations) | Hospital | Outcome | Clinical Outcome | 3 |
| Case fatality (early fatality <24hrs, ratios = for serious, emergency conditions for which a well-performing EUCS could improve chances of survival/ rate = between all contacts with EUCS services for serious emergency or urgent conditions, that die within 7 days, by defined comparators.) | Hospital | Outcome | Clinical Outcome | 3 |
| Mortality in ED | Hospital | Outcome | Clinical Outcome | 3 |
| Preventable death (#, incidence, potentially salvageable) | Hospital | Outcome | Clinical Outcome | 3 |
| Wound complications (infection, hematomas, dehiscense) | Hospital | Outcome | Clinical Outcome | 3 |
| Respiratory complications (Acute respiratory failure/ARDS) | Hospital | Outcome | Clinical Outcome | 2 |
| Glasgow outcome scale or glasgow outcome scale-extended version | Hospital | Outcome | Clinical Outcome | 2 |
| Hospital acquired pneumonia | Hospital | Outcome | Clinical Outcome | 2 |
| Hospital acquired sepsis | Hospital | Outcome | Clinical Outcome | 2 |
| Incidence of new pressure ulcer (decubitus) | Hospital | Outcome | Clinical Outcome | 2 |
| Mortality/survival rate in pts with serious injury (ISS > 16) | Hospital | Outcome | Clinical Outcome | 2 |
| Preventable trauma death (total, rate) | Hospital | Outcome | Clinical Outcome | 2 |
| Public opinion - quality of service | Hospital | Outcome | Clinical Outcome | 1 |
| Acute renal failure | Hospital | Outcome | Clinical Outcome | 1 |
| Mortality (all-cause) rate before discharge in patients who have undergone a procedure in an operating theatre, divided by the total number of procedures, presented as a percentage | Hospital | Outcome | Clinical Outcome | 1 |
| Apiration pneumonia | Hospital | Outcome | Clinical Outcome | 1 |
| C. diff | Hospital | Outcome | Clinical Outcome | 1 |
| Central venous catheter related infection | Hospital | Outcome | Clinical Outcome | 1 |
| Coagulopathy | Hospital | Outcome | Clinical Outcome | 1 |
| Mortality due to haemorrhagic shock | Hospital | Outcome | Clinical Outcome | 1 |
| Mortality >1 h after arrival occur on ward (not in ED) | Hospital | Outcome | Clinical Outcome | 1 |
| Delirum | Hospital | Outcome | Clinical Outcome | 1 |
| DVT | Hospital | Outcome | Clinical Outcome | 1 |
| Extremity compartment syndrome | Hospital | Outcome | Clinical Outcome | 1 |
| Preventable hypoxia or hypotension (incidence) | Hospital | Outcome | Clinical Outcome | 1 |
| Intra-operative arrest | Hospital | Outcome | Clinical Outcome | 1 |
| MI | Hospital | Outcome | Clinical Outcome | 1 |
| Morbidity | Hospital | Outcome | Clinical Outcome | 1 |
| Nonunion of fracture | Hospital | Outcome | Clinical Outcome | 1 |
| Osteomyelitis | Hospital | Outcome | Clinical Outcome | 1 |
| Patient satisfaction | Hospital | Outcome | Clinical Outcome | 1 |
| Post-op hemorrhagic shock | Hospital | Outcome | Clinical Outcome | 1 |
| Pulmonary embolism | Hospital | Outcome | Clinical Outcome | 1 |
| Stroke | Hospital | Outcome | Clinical Outcome | 1 |
| Cost of care rendered to injured patients | Hospital | Outcome | Cost/Finance | 4 |
| Marginal cost per life saved | Hospital | Outcome | Cost/Finance | 1 |
| Airway management (mechanical ventilation) | Hospital | Process | Interventions | 7 |
| Management of open fractures | Hospital | Process | Interventions | 6 |
| Amputation (#/% hospitals able to perform) | Hospital | Process | Interventions | 4 |
| Management of closed fractures (#/% hospitals) | Hospital | Process | Interventions | 4 |
| Basic resuscitation | Hospital | Process | Interventions | 3 |
| Cricothyrotomy/tracheostomy | Hospital | Process | Interventions | 2 |
| I&D | Hospital | Process | Interventions | 2 |
| Drainage of osteomyelitis/septic arthritis (#/% hospitals) | Hospital | Process | Interventions | 2 |
| External fixation | Hospital | Process | Interventions | 2 |
| Fracture immobilization | Hospital | Process | Interventions | 2 |
| Internal fixation (#/% hospitals) | Hospital | Process | Interventions | 2 |
| Non-fixation of femoral diaphyseal fractures in adults | Hospital | Process | Interventions | 2 |
| Skeletal traction | Hospital | Process | Interventions | 2 |
| Chest tube insertion | Hospital | Process | Interventions | 1 |
| Damage control surgery | Hospital | Process | Interventions | 1 |
| Fixation of femoral diaphyseal fractures in adults | Hospital | Process | Interventions | 1 |
| Identification and controll of external bleeding | Hospital | Process | Interventions | 1 |
| Joint dislocation treament | Hospital | Process | Interventions | 1 |
| Open wounds treated and covered | Hospital | Process | Interventions | 1 |
| Prevention/assessment of pressure ulcer | Hospital | Process | Interventions | 1 |
| Skin grafting | Hospital | Process | Interventions | 1 |
| Skin traction | Hospital | Process | Interventions | 1 |
| Immobilize diaphyseal femoral fractures in the Emergency Department | Hospital | Process | Interventions | 1 |
| Stabilize/embolize unstable pelvic fracture | Hospital | Process | Interventions | 1 |
| Quality improvement programs (performance evaluations, patient safety programs) | Hospital | Process | Quality Improvement | 8 |
| Peer review for preventable death | Hospital | Process | Quality Improvement | 2 |
| Readmission rate penalty | Hospital | Process | Quality Improvement | 1 |
| Hospital inspection (e.g., trauma center verification) | Hospital | Process | Quality Improvement | 1 |
| Supply chain improvement | Hospital | Process | Quality Improvement | 1 |
| ED length of stay (>6 hours, < 1 h for pts with GCS < 9 or intubated, < 4 hrs pts ISS > 15, < 2 hrs, trauma room) | Hospital | Process | Quality of Care | 9 |
| Hospital length of stay (for severely injured ISS > 15, >= 10 days for ISS >= 10) | Hospital | Process | Quality of Care | 7 |
| Suspected spine injuries are immobilized (c-collar, logrolled) | Hospital | Process | Quality of Care | 4 |
| Open fx surgery within <12/<8/<6 hours after ED arrival (long bone) | Hospital | Process | Quality of Care | 4 |
| Time to surgery | Hospital | Process | Quality of Care | 4 |
| Airway secured in ED for GCS < 9) | Hospital | Process | Quality of Care | 3 |
| Delays in diagnostic evaluations (All injuries noted within 24 hrs of admission) | Hospital | Process | Quality of Care | 3 |
| Error/Miss in diagnosis | Hospital | Process | Quality of Care | 3 |
| ICU length of stay (severely injured ISS > 15) | Hospital | Process | Quality of Care | 3 |
| Delays in treatment | Hospital | Process | Quality of Care | 3 |
| Discharge planning (assessment including mobility and environmental check, disposition, instruction) | Hospital | Process | Quality of Care | 3 |
| Activation of trauma system (% severely injured that activate trauma team) | Hospital | Process | Quality of Care | 2 |
| Completion of documentation in ED and ICU (hourly) | Hospital | Process | Quality of Care | 2 |
| Obtain sufficient history (Social/Functional) | Hospital | Process | Quality of Care | 2 |
| Physical exame perfromed (Pt undressed, fully examined, and covered for privacy within 30 min of arrival) | Hospital | Process | Quality of Care | 2 |
| Return to ICU (except for ICD9 codes 81.04, 03.53) | Hospital | Process | Quality of Care | 2 |
| Time to arrival of consultant (>30 mins after patient arrival) | Hospital | Process | Quality of Care | 2 |
| Time to arrival of trauma surgeon (>5 mins after patient arrival) | Hospital | Process | Quality of Care | 2 |
| Error in management (Failure to rescue (mortality after the development of a sentinel complication)) | Hospital | Process | Quality of Care | 2 |
| Long bone fxs reduce w/ analgesia and/or splinted within 4 hrs of admission or prior to transfer | Hospital | Process | Quality of Care | 2 |
| Pelvic binder placed for pts with suspected unstable pelvic fracture on hospital arrival | Hospital | Process | Quality of Care | 2 |
| Reduce dislocation of major joint in <1 h/4hrs | Hospital | Process | Quality of Care | 2 |
| Blood products administered appropriately (blood products given before > 3 L of crystalloids have been provided) | Hospital | Process | Quality of Care | 2 |
| Hypothermia monitoring | Hospital | Process | Quality of Care | 2 |
| Vital sign monitoring (within 15 mins of arrival) | Hospital | Process | Quality of Care | 2 |
| Blunt compound tibial fractures treated within 8 hrs of arrival | Hospital | Process | Quality of Care | 2 |
| Time from arrival/admission to surgery (femur) <24/48 h | Hospital | Process | Quality of Care | 2 |
| Time from arrival/admission to surgery (compound fx) <48, 2 | Hospital | Process | Quality of Care | 2 |
| Time to operating room (>2h after patient arrival for life threatening injury, >6h for limb threatening injury) | Hospital | Process | Quality of Care | 2 |
| Breathing assessment within 15 min of arrival | Hospital | Process | Quality of Care | 1 |
| Chest x-ray performed | Hospital | Process | Quality of Care | 1 |
| GCS < X (9-14) and CT scan of head with X (1-4 hrs) of arrival | Hospital | Process | Quality of Care | 1 |
| Hours from admission to preoperative radiographs | Hospital | Process | Quality of Care | 1 |
| Radiology time < 2hrs | Hospital | Process | Quality of Care | 1 |
| Suspected spine injuries are given x-ray | Hospital | Process | Quality of Care | 1 |
| Admitted by surgeon | Hospital | Process | Quality of Care | 1 |
| Consent/patient informed | Hospital | Process | Quality of Care | 1 |
| Pts admitted to surgical service | Hospital | Process | Quality of Care | 1 |
| Transfer of spinal injury patients to acute spinal center | Hospital | Process | Quality of Care | 1 |
| Time to rehab consultation | Hospital | Process | Quality of Care | 1 |
| Head of bed elevated to 45 degree if AVPU is not "A" and pt is not in shock | Hospital | Process | Quality of Care | 1 |
| Time from admission to spica cast application | Hospital | Process | Quality of Care | 1 |
| Time to mobilization after surgery | Hospital | Process | Quality of Care | 1 |
| Patient transfered to another health care facility after spending > 6hrs at initial hospital | Hospital | Process | Quality of Care | 1 |
| Time from surgery to discharge | Hospital | Process | Quality of Care | 1 |
| Open fx receive IV antimicrobials within 1 hr | Hospital | Process | Quality of Care | 1 |
| Pharmacological pain mangement within 30 mins of arrival | Hospital | Process | Quality of Care | 1 |
| 2 large bore IVs within 15 min | Hospital | Process | Quality of Care | 1 |
| Proper fluid administration | Hospital | Process | Quality of Care | 1 |
| Time to hemorrhage control | Hospital | Process | Quality of Care | 1 |
| Venous line established on hospital arrival | Hospital | Process | Quality of Care | 1 |
| Open lacerations of joints treated within 8 hrs of arrival | Hospital | Process | Quality of Care | 1 |
| Hip fracture surgery within 48 hours | Hospital | Process | Quality of Care | 1 |
| Time to treat spine injury: 6 hrs | Hospital | Process | Quality of Care | 1 |
| Time to treatment of compound tibial fractures: 8 hrs | Hospital | Process | Quality of Care | 1 |
| Time from arrival/admission to surgery (open tibia fractures) (within 6 and 24 hours) | Hospital | Process | Quality of Care | 1 |
| Assessment for spinal cord injuries with escalation of red flags | Hospital | Process | Quality of Care | 1 |
| Assessment of malnutrition | Hospital | Process | Quality of Care | 1 |
| DVT risk assessment | Hospital | Process | Quality of Care | 1 |
| Emergency Department initial management | Hospital | Process | Quality of Care | 1 |
| Neurological assessment made with AVPU | Hospital | Process | Quality of Care | 1 |
| Specialist falls assessment | Hospital | Process | Quality of Care | 1 |
| Continuing medical education (esp. trauma) | Hospital | Structure | Education | 6 |
| Formal trauma/emergency training and education | Hospital | Structure | Education | 6 |
| Trauma fellowship training (MSK) | Hospital | Structure | Education | 4 |
| Residency programs | Hospital | Structure | Education | 4 |
| Accredited trauma specific education or training for health providers | Hospital | Structure | Education | 2 |
| % of providers fellowship trained | Hospital | Structure | Education | 1 |
| Duration of medical training to treat MSK trauma | Hospital | Structure | Education | 1 |
| Hospital teaching status | Hospital | Structure | Education | 1 |
| Payment of training | Hospital | Structure | Education | 1 |
| University affiliation | Hospital | Structure | Education | 1 |
| Hospital beds (#, budget per bed, mean/median bed capacity, beds/trauma center) | Hospital | Structure | Infrastructure | 7 |
| ORs (mean number per hospital/trauma center, #/% of hospitals, #/% with pulse ox) | Hospital | Structure | Infrastructure | 7 |
| Laboratory (C-reactive protein, ESR, CBC) | Hospital | Structure | Infrastructure | 7 |
| Trauma centers (country, region, number of hospitals with trauma service) | Hospital | Structure | Infrastructure | 6 |
| Presence of ICU | Hospital | Structure | Infrastructure | 5 |
| Emergency Department (access) | Hospital | Structure | Infrastructure | 4 |
| Pediatric-specific trauma care | Hospital | Structure | Infrastructure | 3 |
| Blood bank | Hospital | Structure | Infrastructure | 3 |
| Post-anaesthesia care unit | Hospital | Structure | Infrastructure | 2 |
| Critical care units | Hospital | Structure | Infrastructure | 2 |
| Medical record system | Hospital | Structure | Infrastructure | 2 |
| Military vs. civilian health facility | Hospital | Structure | Infrastructure | 2 |
| Private vs. public health facilities | Hospital | Structure | Infrastructure | 2 |
| Radiological services | Hospital | Structure | Infrastructure | 2 |
| Type of hospital (health center, primary/district, secondary/provincial, tertiary/regional/mission hospitals/nongovernmental hospitals, private) | Hospital | Structure | Infrastructure | 2 |
| Running water | Hospital | Structure | Infrastructure | 1 |
| Electricity | Hospital | Structure | Infrastructure | 1 |
| Operational power generator | Hospital | Structure | Infrastructure | 1 |
| ICU Beds | Hospital | Structure | Infrastructure | 1 |
| 24/7 Trauma room available | Hospital | Structure | Infrastructure | 1 |
| Pediatric specific trauma centers | Hospital | Structure | Infrastructure | 1 |
| Emergency operating room | Hospital | Structure | Infrastructure | 1 |
| Number (%) of hospitals with limb prosthesis manufacturing units | Hospital | Structure | Infrastructure | 1 |
| Number (%) of hospitals with rehabilitation units | Hospital | Structure | Infrastructure | 1 |
| Number of hospitals | Hospital | Structure | Infrastructure | 1 |
| Number/density of orthopaedic specialty centers | Hospital | Structure | Infrastructure | 1 |
| Pediatric specific ICU facilities | Hospital | Structure | Infrastructure | 1 |
| Number (%) of hospitals performing elective orthopaedic surgery | Hospital | Structure | Infrastructure | 1 |
| Designated trauma centers (accreditation, with heirarchy, intergration in trauma system) | Hospital | Structure | Organization/Component | 5 |
| Collaborative clinical services (gen surg, EM, ortho, neuro \| task allocation, simultaneous task completion, leadership) | Hospital | Structure | Organization/Component | 4 |
| Emergency medicine service | Hospital | Structure | Organization/Component | 3 |
| Availability of PT | Hospital | Structure | Organization/Component | 2 |
| Orthogeriatric management | Hospital | Structure | Organization/Component | 2 |
| Pediatric trauma care | Hospital | Structure | Organization/Component | 2 |
| Access to burn care | Hospital | Structure | Organization/Component | 1 |
| Access to spine care | Hospital | Structure | Organization/Component | 1 |
| Availability of OT | Hospital | Structure | Organization/Component | 1 |
| Availability of prosthetic care | Hospital | Structure | Organization/Component | 1 |
| Availability of psychological counseling | Hospital | Structure | Organization/Component | 1 |
| Availability of trauma services (operative care, critical care, ward care after initial resuscitation) | Hospital | Structure | Organization/Component | 1 |
| General surgery service | Hospital | Structure | Organization/Component | 1 |
| Ortho service | Hospital | Structure | Organization/Component | 1 |
| Neuro surgery service | Hospital | Structure | Organization/Component | 1 |
| Supply chain management | Hospital | Structure | Organization/Component | 1 |
| Pathology facilities available | Hospital | Structure | Organization/Component | 1 |
| Professional Surgical Societies (organization, NGOs, conferences etc) | Hospital | Structure | Organization/Component | 1 |
| Trainees with adequate supervision | Hospital | Structure | Organization/Component | 1 |
| Trauma notification system | Hospital | Structure | Organization/Component | 1 |
| Formal Accrediation (public process for trauma centre designation using outside survey teams, levels of care - heirarchy) | Hospital | Structure | Protocols/Guidelines | 12 |
| Triage protocols | Hospital | Structure | Protocols/Guidelines | 10 |
| Evidence based guidelines for various injuries | Hospital | Structure | Protocols/Guidelines | 7 |
| Referral and transfer protocols | Hospital | Structure | Protocols/Guidelines | 7 |
| Clinical trauma protocols (assessment, management, care coordination) | Hospital | Structure | Protocols/Guidelines | 5 |
| Anesthesia and pain management guidelines (sedation, risk assessment) | Hospital | Structure | Protocols/Guidelines | 4 |
| Appropriate triage (Implementation of golden hour concept) | Hospital | Structure | Protocols/Guidelines | 2 |
| Massive transfusion protocol | Hospital | Structure | Protocols/Guidelines | 2 |
| Patient safety strategies | Hospital | Structure | Protocols/Guidelines | 1 |
| Protocol for open fracture management | Hospital | Structure | Protocols/Guidelines | 1 |
| Resource utilization guidelines | Hospital | Structure | Protocols/Guidelines | 1 |
| Resuscitation guidelines (airway control/ventilation, cervical stabilization, IV access, patient assessment, measurement of vital signs, radiographic investigation) | Hospital | Structure | Protocols/Guidelines | 1 |
| Trauma activation protocol | Hospital | Structure | Protocols/Guidelines | 1 |
| UK's best practice Tariff Indicators | Hospital | Structure | Protocols/Guidelines | 1 |
| Trauma outcomes research | Hospital | Structure | Research | 1 |
| Internal fixation supplies (Implants such as plates and screw sets, nails, prostheses (avalability, affordability (SIGN, fundings sources, cost of implants), number/% of hospitals with a sustainable supply nearly all the time/some of the time) | Hospital | Structure | Resources | 7 |
| X-ray machines (mean number per hospital, number/% of hospitals/facilities) | Hospital | Structure | Resources | 6 |
| CT (access, mean number, total, number/% of hospitals/facilities) | Hospital | Structure | Resources | 5 |
| C-Arms (mean number per hospital, number/% of facilities) | Hospital | Structure | Resources | 5 |
| Chest tubes | Hospital | Structure | Resources | 3 |
| Blood products available | Hospital | Structure | Resources | 3 |
| Immobilization and other equipment available (slings/splints) | Hospital | Structure | Resources | 3 |
| Antibiotics (prophylactic, essential) | Hospital | Structure | Resources | 2 |
| Oxygen | Hospital | Structure | Resources | 2 |
| Pelvic Binder | Hospital | Structure | Resources | 2 |
| Anaesthesia: General inhalational | Hospital | Structure | Resources | 2 |
| Anaesthesia: ketamine | Hospital | Structure | Resources | 2 |
| Anaesthesia: Regional | Hospital | Structure | Resources | 2 |
| Anaesthesia: Spinal | Hospital | Structure | Resources | 2 |
| Bag mask valve | Hospital | Structure | Resources | 2 |
| Fluids | Hospital | Structure | Resources | 2 |
| Pulse oximeter (% of Ors with pulse ox) | Hospital | Structure | Resources | 2 |
| Foley placement | Hospital | Structure | Resources | 1 |
| ABG | Hospital | Structure | Resources | 1 |
| Tetanus vaccination | Hospital | Structure | Resources | 1 |
| Peripheral catheters | Hospital | Structure | Resources | 1 |
| Analgesics | Hospital | Structure | Resources | 1 |
| C-collar | Hospital | Structure | Resources | 1 |
| Suction tubes | Hospital | Structure | Resources | 1 |
| Fracture table | Hospital | Structure | Resources | 1 |
| Anticoagulation | Hospital | Structure | Resources | 1 |
| MRI | Hospital | Structure | Resources | 1 |
| External fixation supplies | Hospital | Structure | Resources | 1 |
| Skeletal traction supplies | Hospital | Structure | Resources | 1 |
| Skin traction supplies | Hospital | Structure | Resources | 1 |
| Spine board | Hospital | Structure | Resources | 1 |
| Tourniquets (availability) | Hospital | Structure | Resources | 1 |
| Endotracheal tube | Hospital | Structure | Resources | 1 |
| Examination gloves | Hospital | Structure | Resources | 1 |
| Eye protection | Hospital | Structure | Resources | 1 |
| Functioning anaesthesia machine | Hospital | Structure | Resources | 1 |
| IV infusion sets | Hospital | Structure | Resources | 1 |
| NG tubes | Hospital | Structure | Resources | 1 |
| Number (%) of hospitals with adequate orthopaedic instruments to treat orthopaedic trauma | Hospital | Structure | Resources | 1 |
| Prophylactic medication avialable | Hospital | Structure | Resources | 1 |
| Resources for airway mangement | Hospital | Structure | Resources | 1 |
| Resources for breathing management | Hospital | Structure | Resources | 1 |
| Resources for circulation and shock management | Hospital | Structure | Resources | 1 |
| Resources for diagnostic and monitoring of trauma patient | Hospital | Structure | Resources | 1 |
| Resources for infection control | Hospital | Structure | Resources | 1 |
| Resources for management of burns and wounds | Hospital | Structure | Resources | 1 |
| Resources for management of extremity injuuries | Hospital | Structure | Resources | 1 |
| Resources for management of head/brain/neck injuries | Hospital | Structure | Resources | 1 |
| Resources for management of spine injuries | Hospital | Structure | Resources | 1 |
| Resources for pain control | Hospital | Structure | Resources | 1 |
| Sterilizing equipment | Hospital | Structure | Resources | 1 |
| Dedicated trauma teams/service (Multidisciplinary) | Hospital | Structure | Staff | 7 |
| Surgeons (Mean/Median number per facility, number/% of hospitals with at least one, density per 100,00, per capita, per trauma center) | Hospital | Structure | Staff | 6 |
| Orthopaedic surgeons (Mean/Median number per facility, number/% of hospitals with at least one) | Hospital | Structure | Staff | 5 |
| Anaesthetic non-physician (Mean/Median number per facility, number/% of hospitals with at least one) | Hospital | Structure | Staff | 4 |
| Orthopaedic clinical officers (Mean/Median number per facility, number/% of hospitals with at least one) | Hospital | Structure | Staff | 4 |
| Trauma-trained physicians (# of, y/n) | Hospital | Structure | Staff | 3 |
| Anaesthesiologist (Mean/Median number per facility, number/% of hospitals with at least one) | Hospital | Structure | Staff | 3 |
| Other doctors providing orthopaedic (Mean/Median number per facility, number/% of hospitals with at least one) | Hospital | Structure | Staff | 3 |
| Physiotherapists (Mean/Median number per facility, number/% of hospitals with at least one) | Hospital | Structure | Staff | 3 |
| Rehabilitation technicians (Mean/Median number per facility, number/% of hospitals with at least one) | Hospital | Structure | Staff | 3 |
| Specialty care availability: orthopedics, radiology, neurosurgery | Hospital | Structure | Staff | 3 |
| Trauma-trained providers (# of, y/) | Hospital | Structure | Staff | 2 |
| Nonphysician clinicians (paramedics/midwives/clinical officers) | Hospital | Structure | Staff | 2 |
| Presence of 24-hour trauma provider coverage (physician in house) | Hospital | Structure | Staff | 2 |
| Healthcare providers (# of) | Hospital | Structure | Staff | 1 |
| Hospital employees (# of) | Hospital | Structure | Staff | 1 |
| MSK residents trained per 1 million (# of) | Hospital | Structure | Staff | 1 |
| Density of specialist surgical providers | Hospital | Structure | Staff | 1 |
| Emergency medicine staff | Hospital | Structure | Staff | 1 |
| General doctor providing anaesthesia | Hospital | Structure | Staff | 1 |
| General doctor providing surgery | Hospital | Structure | Staff | 1 |
| Number of specialist surgical, anesthetic, and obstetric physicians who are working, per 100,000 population | Hospital | Structure | Staff | 1 |
| Number of surgeons caring for MSK trauma per 1 million | Hospital | Structure | Staff | 1 |
| Nurses/100,000 | Hospital | Structure | Staff | 1 |
| Physicians/100,000 | Hospital | Structure | Staff | 1 |
| Ratio of anaesthetists to surgeons | Hospital | Structure | Staff | 1 |
| Surgical residents available | Hospital | Structure | Staff | 1 |
| Surgical technician | Hospital | Structure | Staff | 1 |
| Disability cost coverage/compensation | Posthospital | Equity | Access | 2 |
| Compensation for acute healthcare cost | Posthospital | Equity | Access | 1 |
| Compensation for lifetime healthcare costs | Posthospital | Equity | Access | 1 |
| Quality of life | Posthospital | Outcome | Clinical Outcome | 7 |
| Functional ability | Posthospital | Outcome | Clinical Outcome | 4 |
| Functional: return to work (1/2 years) | Posthospital | Outcome | Clinical Outcome | 4 |
| Functional Independence Measure | Posthospital | Outcome | Clinical Outcome | 2 |
| Functional outcomes: SF-36/12 | Posthospital | Outcome | Clinical Outcome | 2 |
| Avertable fracture DALYs | Posthospital | Outcome | Clinical Outcome | 1 |
| Balance and mobility | Posthospital | Outcome | Clinical Outcome | 1 |
| Development of secondary conditions or injuries | Posthospital | Outcome | Clinical Outcome | 1 |
| Disability | Posthospital | Outcome | Clinical Outcome | 1 |
| Quality of life: EQ-5D | Posthospital | Outcome | Clinical Outcome | 1 |
| Fracture non-union | Posthospital | Outcome | Clinical Outcome | 1 |
| Functional capacity index | Posthospital | Outcome | Clinical Outcome | 1 |
| Functional outcome: mobility subscale of the musculoskeletal function assessment | Posthospital | Outcome | Clinical Outcome | 1 |
| Functional outcome: revised center for epidemiological studies depression scale | Posthospital | Outcome | Clinical Outcome | 1 |
| GOS-E at 6 and 12 months post injury | Posthospital | Outcome | Clinical Outcome | 1 |
| Long-term physical disability | Posthospital | Outcome | Clinical Outcome | 1 |
| Mortality (postdischarge) | Posthospital | Outcome | Clinical Outcome | 1 |
| Mortality over 1, 2 and 3 years | Posthospital | Outcome | Clinical Outcome | 1 |
| Observe, care taker, or dependant consequences | Posthospital | Outcome | Clinical Outcome | 1 |
| Pain and discomfort | Posthospital | Outcome | Clinical Outcome | 1 |
| Psychological disability | Posthospital | Outcome | Clinical Outcome | 1 |
| Quality of Wellbeing Scale | Posthospital | Outcome | Clinical Outcome | 1 |
| Short Musculoskeletal Function Assessment | Posthospital | Outcome | Clinical Outcome | 1 |
| Visual Analog Scale for pain | Posthospital | Outcome | Clinical Outcome | 1 |
| Intangible costs | Posthospital | Outcome | Cost/Finance | 1 |
| Tangible cost | Posthospital | Outcome | Cost/Finance | 1 |
| Systematic pain assessment | Posthospital | Process | Interventions | 1 |
| % of pts discharged directly home vs. rehab facility | Posthospital | Process | Quality of Care | 1 |
| Access to rehab | Posthospital | Structure | Infrastructure | 11 |
| Access to post-hospital institutional care | Posthospital | Structure | Infrastructure | 2 |
| Posthospital care/rehab | Posthospital | Structure | Organization/Component | 2 |
| Referrals for further management | Posthospital | Structure | Organization/Component | 1 |
| Rehab protocols | Posthospital | Structure | Protocols/Guidelines | 1 |
| The lead agency has incorporated, within the trauma system plan and the trauma center standards, requirements for rehabilitation services, including interfacility transfer of trauma patients to rehabilitation centers. | Posthospital | Structure | Protocols/Guidelines | 1 |
| Resources for rehabilitiation | Posthospital | Structure | Resources | 1 |
| Caregiver support and availability | Posthospital | Structure | Staff | 1 |
| Multi-professional rehab team | Posthospital | Structure | Staff | 1 |
| Post-discharge psych | Posthospital | Structure | Staff | 1 |
| Post-discharge PT/OT | Posthospital | Structure | Staff | 1 |
| Post-discharge rehab | Posthospital | Structure | Staff | 1 |
| Transportation methods (private vs. public transport, formalized vehicle access, …) | Prehospital | Equity | Access | 2 |
| % of population with access to prehospital care | Prehospital | Equity | Access | 1 |
| Access to injury care - care in the battlefield | Prehospital | Equity | Access | 1 |
| Climate - seasonal variation in road access | Prehospital | Equity | Access | 1 |
| Transportation to a TC from the scene of accident (direct access) or from a non-designated hospital (indirect access) | Prehospital | Equity | Access | 1 |
| Prehospital mortality | Prehospital | Outcome | Clinical Outcome | 6 |
| Shock rate (systolic blood pressure (SBP) <90 mmHg) | Prehospital | Outcome | Clinical Outcome | 1 |
| Prehospital airway control (intubation, endotracheal intubation) | Prehospital | Process | Interventions | 5 |
| Immobilization of injuries | Prehospital | Process | Interventions | 2 |
| Prehospital triage | Prehospital | Process | Interventions | 2 |
| Ability to safely extricate people from scene | Prehospital | Process | Interventions | 1 |
| ALS/BLS at scene | Prehospital | Process | Interventions | 1 |
| Bleeding control at scene | Prehospital | Process | Interventions | 1 |
| Pre-hospital IV placement | Prehospital | Process | Interventions | 1 |
| Prehospital BP monitoring | Prehospital | Process | Interventions | 1 |
| Prehospital quality improvement | Prehospital | Process | Quality Improvement | 2 |
| Prehospital triage criteria are regularly evaluated and updated to ensure acceptable rates of sensitivity and specificity for appropriately identifying the major trauma patient. and systemdefined | Prehospital | Process | Quality Improvement | 1 |
| EMS system has adequate medical oversight | Prehospital | Process | Quality Improvement | 1 |
| Prehospital time (Total) (to trauma center, timeliness: within 60 mins) | Prehospital | Process | Quality of Care | 12 |
| Emergency services/BLS response time (<20 mins, mean response time) | Prehospital | Process | Quality of Care | 8 |
| Transport times (mean, total time) | Prehospital | Process | Quality of Care | 3 |
| Scene time (10 - 30 mins) | Prehospital | Process | Quality of Care | 3 |
| Time from injury to admission (femur fx, open tibia fracture within 6 or 24 hrs) | Prehospital | Process | Quality of Care | 2 |
| Estimated proportion of seriously injured patients transported by ambulance | Prehospital | Process | Quality of Care | 1 |
| Field airway inadequacy | Prehospital | Process | Quality of Care | 1 |
| Health facility notified prior to arrival of patient | Prehospital | Process | Quality of Care | 1 |
| Injury severity categorized | Prehospital | Process | Quality of Care | 1 |
| Patients with ground scene time/air scene time/combine ground and air >=20 mins | Prehospital | Process | Quality of Care | 1 |
| Presence of the medical report in the prehospital phase | Prehospital | Process | Quality of Care | 1 |
| Arrival time to time Life Flight notified >=20 mins | Prehospital | Process | Quality of Care | 1 |
| Prehospital staff trained in trauma (first responders/EMS in basic trauma care, ATLS, PHTLS) | Prehospital | Structure | Education | 11 |
| Modes of prehospital transport (ambulance, helicopter, plane) | Prehospital | Structure | Infrastructure | 5 |
| Ambulances (Presences/#) | Prehospital | Structure | Infrastructure | 4 |
| # of ambulance dispatch stations | Prehospital | Structure | Infrastructure | 1 |
| Prehopsital trauma care | Prehospital | Structure | Organization/Component | 9 |
| Emergency response number or call center available to public | Prehospital | Structure | Organization/Component | 7 |
| EMS system present (care provided: BLS vs. ALS, types of procedures performed, health professionals staffing ambulance) | Prehospital | Structure | Organization/Component | 6 |
| Presence of prehospital system | Prehospital | Structure | Organization/Component | 5 |
| Existence of ambulance system and pre-hospital team | Prehospital | Structure | Organization/Component | 4 |
| Established dispatch systems | Prehospital | Structure | Organization/Component | 2 |
| Prehospital notificaiton system (advanced notification of hospital of injured patients) | Prehospital | Structure | Organization/Component | 2 |
| Trauma communication center | Prehospital | Structure | Organization/Component | 1 |
| Established triage criteria which permit bypassing non-trauma centers to deliver patients to trauma centers | Prehospital | Structure | Protocols/Guidelines | 5 |
| Prehospital dispatch protocols (time standards for arrival on scene) | Prehospital | Structure | Protocols/Guidelines | 3 |
| Prehospital guidelines (management/transfer/triage) | Prehospital | Structure | Protocols/Guidelines | 2 |
| Protocols or guidelines for prehospital care | Prehospital | Structure | Protocols/Guidelines | 2 |
| Fluid available in the prehospital setting | Prehospital | Structure | Resources | 3 |
| O2 therapy in prehospital setting | Prehospital | Structure | Resources | 1 |
| Formally trauma trained prehospital providers (paramedics, first responders) | Prehospital | Structure | Staff | 3 |
| Incorporation of lay providers into trauma care system (community and police) | Prehospital | Structure | Staff | 3 |
| Network of community leaders/police/commercial drivers/volunteers with basic first aid | Prehospital | Structure | Staff | 2 |
| Level of healthcare provider on ambulance | Prehospital | Structure | Staff | 1 |
| Road traffic injuries | Prevention | Equity | Burden | 3 |
| Alcohol/drug related injuries | Prevention | Equity | Burden | 1 |
| Burden of preventable disease due to lack of adequate acute care services | Prevention | Equity | Burden | 1 |
| Burden of industrial injuries | Prevention | Equity | Burden | 1 |
| Burden of agriculture injuries | Prevention | Equity | Burden | 1 |
| Fatal accident rate/km traveled | Prevention | Equity | Burden | 1 |
| Femoral shaft fracture from road traffic collisions per year | Prevention | Equity | Burden | 1 |
| Interpersonal violence | Prevention | Equity | Burden | 1 |
| Mechanism of non-fatal injury | Prevention | Equity | Burden | 1 |
| Mechanism of non-natural mortalities | Prevention | Equity | Burden | 1 |
| Mechanisms of injury | Prevention | Equity | Burden | 1 |
| Road traffic accidents | Prevention | Equity | Burden | 1 |
| Substance abuse | Prevention | Equity | Demographics | 1 |
| RTI related deaths (mortality per 100,000 persons) | Prevention | Outcome | Clinical Outcome | 2 |
| Alcohol screening | Prevention | Process | Interventions | 1 |
| Discharge on bone protection medication | Prevention | Process | Interventions | 1 |
| Future fracture prevention assessment | Prevention | Process | Interventions | 1 |
| Road infrastructure | Prevention | Structure | Infrastructure | 1 |
| Injury Prevention Efforts (committees, initiatives) | Prevention | Structure | Organization/Component | 5 |
| National injury surveillance system (population-based public health surveillance, and evaluation, for acute and chronic traumatic injury and injury prevention) | Prevention | Structure | Organization/Component | 5 |
| WHO CORE Indicator Health Security | Prevention | Structure | Organization/Component | 1 |
| Screening and preventative care | Prevention | Structure | Organization/Component | 1 |
| Road safety measures (legislation on blood alcohol, speed limits, seat belts, helmets for bike and motorcycles, child safety laws) | Prevention | Structure | Protocols/Guidelines | 5 |
| Prevention strategies (legislation protecting individual rights, gun control, alcohol/substance abuse, ...) | Prevention | Structure | Protocols/Guidelines | 2 |
| Osteoporosis testing/medications | Prevention | Structure | Resources | 1 |
