# Supplementary material for: Indicators for the evaluation of musculoskeletal trauma systems: A scoping review and Delphi study
Source: PLoS One. 2023 Aug 31;18(8):e0290816. doi: 10.1371/journal.pone.0290816 (PMC10470913; doi:10.1371/journal.pone.0290816)
Supplement: S5 File — (DOCX) [file pone.0290816.s005.docx]

Supporting Information 5: Expanded classification of unique performance indicators identified in study.

|  | General | Prevention | Pre-Hospital | Hospital | Post-Hospital | Total |
| --- | --- | --- | --- | --- | --- | --- |
| Structure | 37 | 8 | 22 | 152 | 12 | 221 |
| Organization/  Component | 22 | 4 | 8 | 20 | 2 | 56 |
| Protocols/  Guidelines | 3 | 2 | 4 | 14 | 2 | 25 |
| Infrastructure | - | 1 | 3 | 28 | 2 | 34 |
| Staff | 3 | - | 4 | 28 | 5 | 40 |
| Resources | 2 | 1 | 2 | 51 | 1 | 57 |
| Education | 5 | - | 1 | 10 | - | 16 |
| Research | 2 | - | - | 1 | - | 3 |
| Process | 9 | 3 | 23 | 91 | 2 | 128 |
| Interventions | - | 3 | 8 | 24 | 1 | 36 |
| Quality Improvement | 4 | - | 3 | 5 | - | 12 |
| Quality of Care | 5 | - | 12 | 62 | 1 | 80 |
| Outcome | 13 | 1 | 2 | 42 | 26 | 84 |
| Cost/Finance | 6 | - | - | 2 | 2 | 10 |
| Clinical Outcome | 7 | 1 | 2 | 40 | 24 | 74 |
| Equity | 50 | 13 | 5 | 28 | 3 | 99 |
| Trauma-related burden | 11 | 12 | - | 12 | - | 35 |
| Demographics | 8 | 1 | - | - | - | 9 |
| Access | 9 | - | 5 | 16 | 3 | 33 |
| Non-trauma burden | 10 | - | - | - | - | 10 |
| Economics | 12 | - | - | - | - | 12 |
| Total | 109 | 25 | 52 | 303 | 43 | 498 |
